# Stage IV non-small cell lung cancer among young individuals: Incidence, presentations, and survival outcomes of conventional therapies

**DOI:** 10.3389/fonc.2022.894780

**Published:** 2022-11-11

**Authors:** Jing-Sheng Cai, Man-Tang Qiu, Fan Yang, Xun Wang

**Affiliations:** Department of Thoracic Surgery, Peking University People’s Hospital, Beijing, China

**Keywords:** stage IV, non-small cell lung cancer, age ≤ 45 years, incidence, outcome

## Abstract

**Background:**

There is a paucity of data published on the clinicopathological features and prognosis of stage IV non-small cell lung cancer (NSCLC) patients aged ≤45 years. Herein, we evaluated a large clinical series in an effort to provide a clearer picture of this population.

**Methods:**

The least absolute shrinkage and selection operator (LASSO)-penalized Cox regression model was performed to identify prognostic factors for NSCLC among individuals aged ≤45 years. The Kaplan–Meier method with log-rank test was used to compare overall survival (OS) differences between groups. Competing risk analysis with the Fine–Gray test was used to analyze cancer-specific survival (CSS) differences. Propensity score matching (PSM) was used to minimize selection bias.

**Results:**

Incidence-rate analyses, including 588,680 NSCLC cases (stage IV, 233,881; age ≤ 45 years stage IV, 5,483; and age > 45 years stage IV, 228,398) from 2004 to 2015, showed that the incidence of stage IV NSCLC among young individuals decreased over the years. In comparative analyses of clinical features and survival outcomes, a total of 48,607 eligible stage IV cases (age ≤ 45 years stage IV, 1,390; age > 45 years stage IV, 47,217) were included. The results showed that although patients in the young cohort were more likely to be diagnosed at advanced stages, they were also more likely to receive aggressive treatments. In addition, the survival rates of the young patients were superior to those of the older patients both before and after PSM.

**Conclusions:**

Stage IV NSCLC patients aged ≤45 years comprise a relatively small but special NSCLC subgroup. Although this population had better survival outcomes than older patients, these patients deserve more attention due to their young age and the significant socioeconomic implications.

## Introduction

Lung cancer is a serious global pandemic (1, 2). Non-small cell lung cancer (NSCLC) accounts for approximately 85% of all lung cancer cases. Approximately 40% of NSCLC patients are initially diagnosed at stage IV (3). Because the median age at diagnosis is 70 years (4), NSCLC is often regarded as a disease among older people. However, over the past few decades, the incidence of NSCLC in young individuals has been increasing gradually (5–7). Given the substantial societal and economic effects of NSCLC, more in-depth investigations are needed to examine this disease among young patients (8).

Previous clinical series demonstrated that young NSCLC patients are more likely to be female, to be non-smokers, to have adenocarcinoma (ADC) subtypes, and to have advanced-stage diseases than older NSCLC patients (4, 5, 9–16). Furthermore, the survival rate of young patients is inferior to that of older patients (9–11, 13). However, these studies focused on the entire entity of young NSCLC patients, and no literature is available about the clinicopathological features and prognosis of young stage IV NSCLC patients.

Given the paucity of related studies, we sought to better understand stage IV NSCLC among young patients (age ≤ 45 years) by analyzing the data deposited in the Surveillance, Epidemiology, and End Results (SEER) Program with the purpose of sketching an outline of this population.

## Materials and methods

### Included subjects

**Figure 1:**
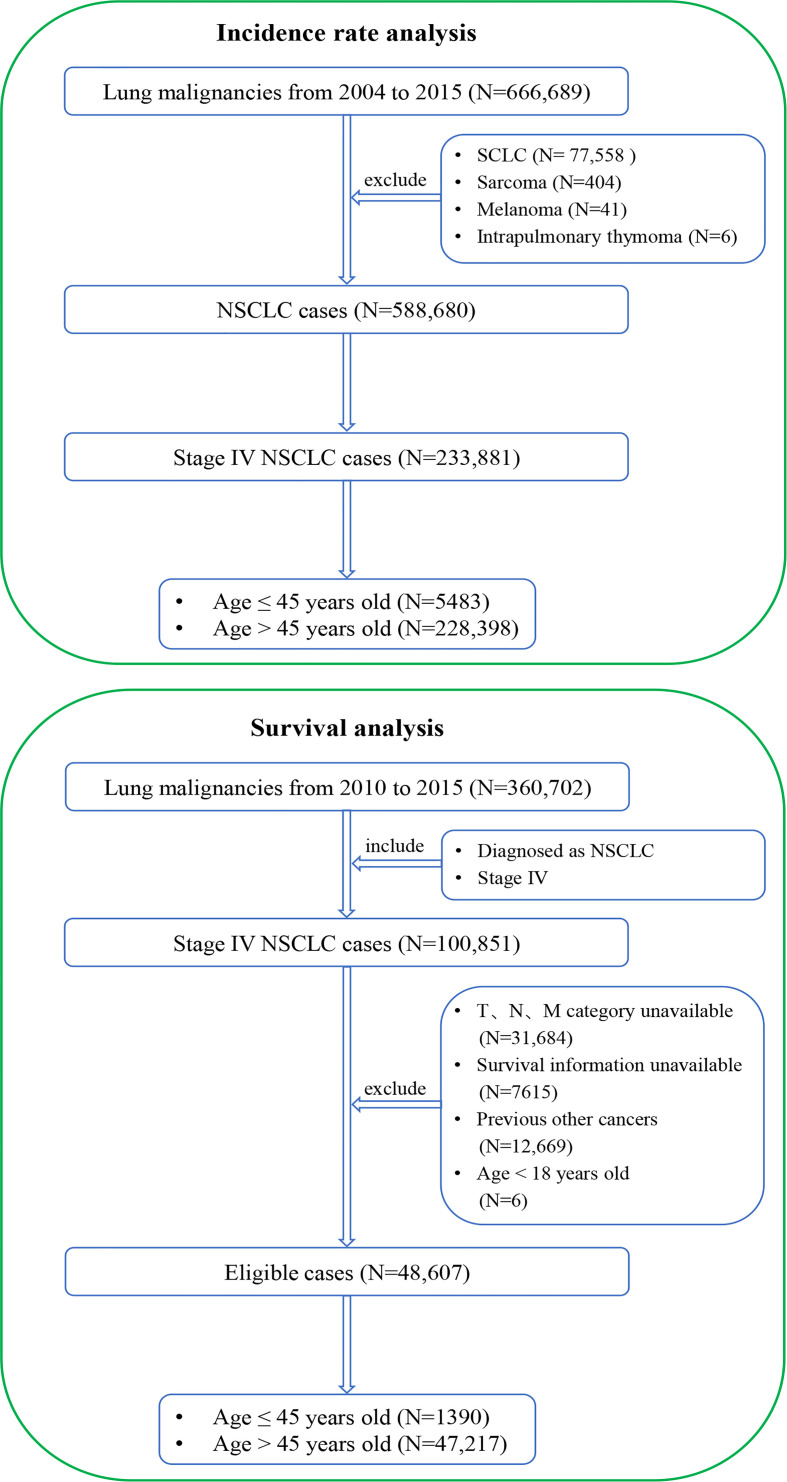
Flowcharts of patient selection. The detailed selection process of stage IV NSCLC patients from 2004 to 2015 for incidence-rate analysis (top) and the detailed selection process of stage IV NSCLC patients from 2010 to 2015 for presentations and survival outcomes analysis (bottom). NSCLC, non-small cell lung cancer; SCLC, small cell lung cancer.

To analyze the incidence rate, lung cancer patients from 2004 to 2015 were extracted from the SEER database (https://seer.cancer.gov/). The inclusion criterion was a diagnosis of lung malignancy. The exclusion criteria were 1) lung tumors other than NSCLC and 2) stage I–III diseases (the 8th edition of the tumor–node–metastasis [TNM] staging system (17)). The flowchart of patient selection is shown in **Figure 1**.

To analyze clinicopathological features and survival outcomes, lung cancer patients from 2010 to 2015 were extracted from the SEER database. The inclusion criteria were as follows: 1) pathologically diagnosed as NSCLC and 2) stage IV diseases. The exclusion criteria were as follows: 1) unavailable TNM stage information, 2) unavailable survival information, 3) previous other cancers, and (4) age < 18 years. The eligible stage IV NSCLC patients were categorized into two groups: stage IV NSCLC aged ≤45 years and stage IV NSCLC aged >45 years. The corresponding flowchart of patient selection is shown in **Figure 1**.

Stage IV patients from 2004 to 2015 were included in the incidence-rate analysis because of the large number of cases, which could lead to a more reliable conclusion. Patients from 2010 to 2015 were included in the clinical characteristics and survival outcomes analysis because only the information about specific metastasis sites was available for the patients in this period, which is an important prognostic factor for metastatic NSCLC patients.

### Ethics

We obtained permission to access the SEER dataset (reference number 12962-Nov2019) using SEER*Stat software version 8.3.4. The study was conducted in accordance with the Declaration of Helsinki. The Ethics Board of Peking University People’s Hospital approved this study. This was an open database study, and only non-identifiable information was used. Therefore, this study was dispensed with acquiring signed informed consent forms and ethical approval.

### Data collection

This work was supported by the NationalNatural Science funds (grant number82173386).The following anonymized data, including age (continue), sex (male and female), ethnicity (Caucasian, African, and other), marital status (married and other), tumor location (upper lobe [UL], middle lobe [ML], low lobe [LL], and other), surgery (no and yes), chemotherapy (no and yes), radiotherapy (no and yes), grade (well differentiated, moderately differentiated, poorly differentiated, undifferentiated, and unknown), TNM stage (stage IVA, stage IVB, and stage IV), T category (T1, T2, T3, and T4), N category (N0, N1, N2, and N3), M category (M1a, M1b, M1c, and M1), bone metastasis (no and yes), brain metastasis (no and yes), liver metastasis (no and yes), intrapulmonary metastasis (no and yes), cause of death, patient status, and survival time, were retrieved. The current 8th edition of the TNM staging system (17) was used in this study.

### Follow-up

The primary endpoints were overall survival (OS) and cancer-specific survival (CSS). OS was calculated as the time interval from the date of initial diagnosis to the date of death or the date of the last follow-up evaluation. CSS was calculated as the time interval from the date of initial diagnosis to the date of death attributed to NSCLC or the date of the last follow-up evaluation. The survival information, including survival time, survival status, and cause of death, is available in the SEER database. NSCLC patients with an exact survival status and survival time were included, and those with a survival time = 0 months were excluded from this study. The median follow-up time of the entire stage IV cohort, age ≤ 45 years stage IV cohort, and age > 45 years stage IV cohort were 6 months (range, 1–83 months), 12 months (range, 1–83 months), and 6 months (range, 1–83 months), respectively.

### Statistical analysis

R version 3.5.2 (The R Foundation for Statistical Computing, Vienna, Austria; http://www.r-project.org) and IBM SPSS Statistics (version 25.0, IBM Corp, Armonk, NY, USA) were applied to the statistical analysis. The Kaplan–Meier method with log-rank test was used to compare OS differences between groups. Competing risk analysis with the Fine–Gray test (18) was used to compare CSS differences between groups. One-to-two propensity score matching (PSM) (19) between the age ≤ 45 years cohort and the age > 45 years cohort was carried out to minimize bias. The nearest-neighbor matching method with a caliper distance of 0.0001 was used in the PSM algorithm. The variables, including sex, surgery, chemotherapy, radiotherapy, histology, grade, TNM stage, T category, N category, M category, bone metastasis, brain metastasis, liver metastasis, and intrapulmonary metastasis, were included in the PSM algorithm. The variables, including age, sex, ethnicity, marital status, tumor location, surgery, chemotherapy, radiotherapy, histology, grade, TNM stage, T category, N category, M category, bone metastasis, brain metastasis, liver metastasis, and intrapulmonary metastasis, were included in the least absolute shrinkage and selection operator (LASSO) regression model (20), which minimizes the risk of overfitting and further selects the potential prognostic factors. The selected variables were then included in a stepwise multivariable Cox regression analysis to determine the final independent prognostic factors. Statistically significant factors selected from the LASSO-penalized multivariable Cox analysis were used to develop a nomogram (21). The C-index (22) and the receiver operating characteristic (ROC) curves with an area under the curve (AUC) were used to evaluate the performances of the models. Categorical variables were expressed as numbers and percentages and were compared between groups using Pearson’s χ^2^ test. A two-sided *p* < 0.05 was considered statistically significant.

## Results

### Incidence-rate analysis

**Figure 2:**
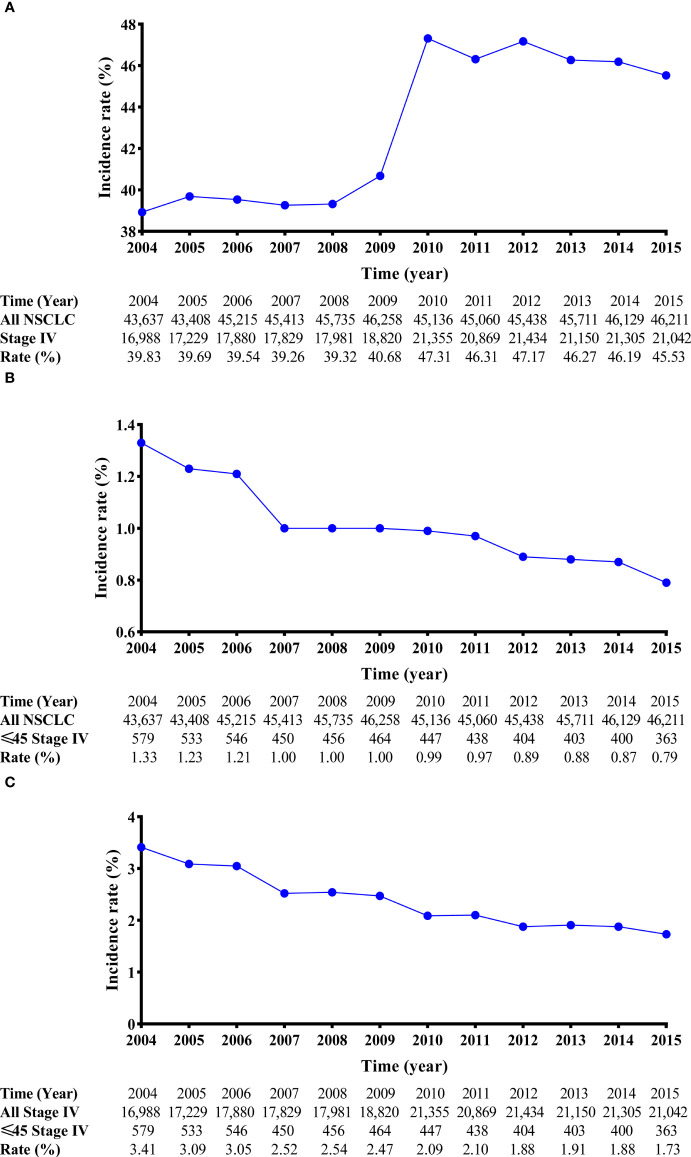
Line charts of the incidence of stage IV NSCLC patients from 2004 to 2015. The incidence-rate analyses of stage IV NSCLC patients in the entire cohort **(A)**. The incidence-rate analyses of stage IV NSCLC patients aged ≤45 years in the entire cohort **(B)**; and the incidence-rate analyses of stage IV NSCLC patients aged ≤45 years in the stage IV NSCLC cohort. **(C)** NSCLC, non-small cell lung cancer.

This work was supported by the NationalNatural Science funds (grant number82173386).From 2004 to 2015, 666,689 cases of lung malignancies were retrospectively reviewed. After the inclusion and exclusion criteria were applied, 233,881 eligible stage IV NSCLC cases (age ≤ 45 years, 5,483 cases; age > 45 years, 228,398 cases) were selected. In the entire cohort, the crude incidence of stage IV diseases was stable between 2004 and 2009 (approximately 40.00%). However, it increased by approximately 6.00% between 2010 and 2015 (**Figure 2A**). Considering the incidence of age ≤ 45 years stage IV NSCLC, a decreasing tendency was observed between 2004 and 2015 (1.33% in 2004 and 0.79% in 2015; **Figure 2B**). In stage IV NSCLC cohort, the incidence of age ≤ 45 years still had an uninterrupted decrease during this period (3.41% in 2004 and 1.73% in 2015; **Figure 2C**).

### Patient characteristics

From 2010 to 2015, the data of 360,702 lung malignancy cases were reviewed. A total of 48,607 eligible stage IV NSCLC cases (age ≤ 45 years, 1,390 cases; age > 45 years, 47,217 cases) were selected. The clinicopathological features are listed in **Table 1**. Regarding the age ≤ 45 years in stage IV NSCLC patients, there was no sex difference (male vs. female = 48.8% vs. 51.2%). The majority of patients were Caucasian (67.9%). Only a small proportion of patients underwent surgery (9.1%) and radiotherapy (21.7%). Most patients underwent chemotherapy (81.5%). ADC was the predominant histological subtype (66.6%). Over half of the cases were diagnosed as stage IVA disease (53.5%). Most of the cases had local/distant lymph node metastasis (N2 category, 46.0%; N3 category, 27.0%). Regarding the metastatic sites, 43.9% of the patients had bone metastasis, 38.6% had brain metastasis, 19.0% had liver metastasis, and 31.3% had intrapulmonary metastasis.

**Table 1:** Baseline features of stage IV NSCLC patients ≤45 years and those >45 years before and after PSM.

Characteristic	Before PSM			After PSM		
	≤45 (N = 1,390)	>45 (N = 47,217)	*p*	≤45 (N = 1,320)	>45 (N = 2,589)	*p*
Sex			<0.001			1.000
Male	679 (48.8)	25,887 (54.8)		652 (49.4)	1,278 (49.4)	
Female	711 (51.2)	21,330 (45.2)		668 (50.6)	1,311 (50.6)	
Surgery			<0.001			0.334
No	1,264 (90.9)	45,124 (95.6)		1,230 (93.2)	2,433 (94.0)	
Yes	126 (9.1)	2,093 (4.4)		90 (6.8)	156 (6.0)	
Chemotherapy			<0.001			0.938
No	257 (18.5)	18,399 (39.0)		242 (18.3)	472 (18.2)	
Yes	1,133 (81.5)	28,818 (61.0)		1,078 (81.7)	2,117 (81.8)	
Radiotherapy			<0.001			0.618
No	1,089 (78.3)	41,643 (88.2)		1,064 (80.6)	2,104 (81.3)	
Yes	301 (21.7)	5,574 (11.8)		256 (19.4)	485 (18.7)	
Histology			<0.001			0.954
ADC	926 (66.6)	26,715 (56.6)		895 (67.8)	1,768 (68.3)	
SCC	125 (9.0)	9,999 (21.2)		116 (8.8)	224 (8.7)	
Other	339 (24.4)	10,503 (22.2)		309 (23.4)	597 (23.1)	
Grade			0.167			0.210
Well	50 (3.6)	1,344 (2.8)		47 (3.6)	95 (3.7)	
Moderately	190 (13.7)	6,618 (14.0)		176 (13.3)	285 (11.0)	
Poorly	387 (27.8)	14,058 (29.8)		364 (27.6)	725 (28.0)	
Undifferentiated	29 (2.1)	770 (1.6)		25 (1.9)	38 (1.5)	
Unknown	734 (52.8)	24,427 (51.7)		708 (53.6)	1,446 (55.9)	
TNM stage			<0.001			0.936
IVA	744 (53.5)	29,315 (62.1)		719 (54.5)	1,426 (55.1)	
IVB	321 (23.1)	7,608 (16.1)		294 (22.3)	568 (21.9)	
IV	325 (23.4)	10,294 (21.8)		307 (23.3)	595 (23.0)	
T category			0.005			0.999
T1	346 (24.9)	10,822 (22.9)		330 (25.0)	650 (25.1)	
T2	412 (29.6)	15,680 (33.2)		404 (30.6)	796 (30.7)	
T3	260 (18.7)	9,410 (19.9)		241 (18.3)	470 (18.2)	
T4	372 (26.8)	11,305 (23.9)		345 (26.1)	673 (26.0)	
N category			<0.001			0.899
N0	266 (19.1)	11,708 (24.8)		255 (19.3)	495 (19.1)	
N1	110 (7.9)	4,119 (8.7)		95 (7.2)	171 (6.6)	
N2	639 (46.0)	21,612 (45.8)		617 (46.7)	1,231 (47.5)	
N3	375 (27.0)	9,778 (20.7)		353 (26.7)	692 (26.7)	
M category			<0.001			0.988
1a	259 (18.6)	12,472 (26.4)		250 (18.9)	495 (19.1)	
1b	485 (34.9)	16,842 (35.7)		469 (35.5)	931 (36.0)	
1c	321 (23.1)	7,608 (16.1)		294 (22.3)	568 (21.9)	
1	325 (23.4)	10,295 (21.8)		307 (23.3)	595 (23.0)	
Bone metastasis			<0.001			0.926
No	780 (56.1)	28,782 (61.0)		751 (56.9)	1,477 (57.0)	
Yes	610 (43.9)	18,435 (39.0)		569 (43.1)	1,112 (43.0)	
Brain metastasis			<0.001			0.732
No	854 (61.4)	33,743 (71.5)		816 (61.8)	1,615 (62.4)	
Yes	536 (38.6)	13,474 (28.5)		504 (38.2)	974 (37.6)	
Liver metastasis			0.097			0.547
No	1,126 (81.0)	39,056 (82.7)		1,085 (82.2)	2,148 (83.0)	
Yes	264 (19.0)	8,161 (17.3)		235 (17.8)	441 (17.0)	
Intrapulmonary metastasis			0.710			0.818
No	955 (68.7)	32,661 (69.2)		913 (69.2)	1,800 (69.5)	
Yes	435 (31.3)	14,556 (30.8)		407 (30.8)	789 (30.5)	

NSCLC, non-small cell lung cancer; PSM, propensity score matching; ADC, adenocarcinoma; SCC, squamous cell carcinoma; TNM, tumor–node–metastasis.

Before PSM, when compared with the age > 45 years stage IV NSCLC cohort, there were more men in the age ≤ 45 years stage IV NSCLC cohort (*p* < 0.001). In addition, more patients received surgery, chemotherapy, and radiotherapy in the age ≤ 45 years stage IV NSCLC cohort (surgery, 9.1% vs. 4.4%, *p* < 0.001; chemotherapy, 81.5% vs. 61.0%, *p* < 0.001; radiotherapy, 21.7% vs. 11.8%, *p* < 0.001). More patients in the age > 45 years stage IV NSCLC cohort suffered from intrathoracic metastasis (62.1% vs. 53.5%, *p* < 0.001). However, they were unlikely to have lymph node metastasis (*p* < 0.001). After PSM, there were 1,320 and 2,589 cases in the young and older patient groups, respectively. The covariates between these two groups were well-balanced (**Table 1**).

### Least absolute shrinkage and selection operator-penalized Cox regression analysis and nomogram

To examine OS, 14 variables, including age, sex, ethnicity, marital status, tumor location, surgery, chemotherapy, histology, grade, T category, N category, M category, bone metastasis, and liver metastasis, were selected using the LASSO model (**Figures S1A, B**). In further analyses, the multivariable Cox regression analysis confirmed that age, ethnicity, surgery, chemotherapy, histology, grade, T category, N category, M category, and bone metastasis were independent prognostic factors (**Table 2**). The corresponding nomogram was developed (**Figure S2A**). The C-index of this nomogram was 0.67 (95% CI: 0.65–0.69). The AUC of the nomogram was 0.69 (**Figure S3A**).

**Table 2:** LASSO-penalized multivariable Cox analysis of the stage IV NSCLC patients ≤45 years.

Characteristic	OS			CSS		
	HR	95% CI	*p*	HR	95% CI	*p*
Age, years	1.026	1.013–1.039	<0.001	1.029	1.015–1.043	<0.001
Sex			0.527			0.586
Male	1			1		
Female	1.040	0.920–1.176		1.036	0.913–1.176	
Ethnicity			<0.001			<0.001
Caucasian	1			1		
African	1.171	0.989–1.387		1.164	0.977–1.387	
Other	0.670	0.561–0.800		0.664	0.553–0.798	
Marital status			0.204			0.485
Married	1			1		
Other	1.085	0.957–1.230		1.047	0.920–1.192	
Tumor location			0.113			0.118
UL	1			1		
ML	0.785	0.594–1.038		0.779	0.583–1.041	
LL	0.863	0.743–1.003		0.867	0.742–1.013	
Other	0.995	0.844–1.174		1.016	0.857–1.204	
Surgery			<0.001			<0.001
No	1			1		
Yes	0.459	0.349–0.604		0.406	0.301–0.549	
Chemotherapy			<0.001			<0.001
No	1			1		
Yes	1.948	1.668–2.275		1.959	1.667–2.302	
Radiotherapy						0.553
No				1		
Yes				1.050	0.893–1.235	
Histology			0.029			0.055
ADC	1			1		
SCC	1.260	1.022–1.552		1.226	0.986–1.524	
Other	1.159	0.999–1.343		1.160	0.996–1.351	
Grade			<0.001			<0.001
Well	1			1		
Moderately	2.109	1.292–3.442		2.114	1.261–3.544	
Poorly	2.533	1.574–4.077		2.608	1.580–4.307	
Undifferentiated	4.025	2.198–7.369		4.194	2.234–7.873	
Unknown	2.241	1.399–3.589		2.240	1.362–3.682	
T category			<0.001			<0.001
T1	1			1		
T2	0.951	0.803–1.128		0.956	0.802–1.139	
T3	1.203	1.000–1.448		1.209	0.998–1.464	
T4	1.423	1.193–1.699		1.426	1.188–1.713	
N category			<0.001			<0.001
N0	1			1		
N1	1.395	1.074–1.811		1.347	1.024–1.772	
N2	1.409	1.179–1.685		1.420	1.180–1.709	
N3	1.625	1.336–1.977		1.633	1.333–2.000	
M category			0.004			<0.001
1a	1			1		
1b	1.110	0.912–1.351		1.347	1.024–1.772	
1c	1.158	0.870–1.540		1.420	1.180–1.709	
1	1.401	1.148–1.709		1.633	1.333–2.000	
Bone metastasis			0.039			0.061
No	1			1		
Yes	1.192	1.009–1.408		1.183	0.992–1.410	
Liver metastasis			0.134			0.116
No	1			1		
Yes	1.149	0.958–1.379		1.164	0.963–1.406	

LASSO, least absolute shrinkage and selection operator; NSCLC, non-small cell lung cancer; PSM, propensity score matching; UL, upper lobe; ML, middle lobe; LL, low lobe; ADC, adenocarcinoma; SCC, squamous cell carcinoma.

To examine CSS, 15 variables, including age, sex, ethnicity, marital status, tumor location, surgery, chemotherapy, radiotherapy, histology, grade, T category, N category, M category, bone metastasis, and liver metastasis, were selected using the LASSO model (**Figures S1C, D**). The multivariable Cox regression analysis confirmed that age, ethnicity, surgery, chemotherapy, grade, T category, N category, and M category were independent prognostic factors (**Table 2**). The corresponding nomogram was established (**Figure S2B**). The C-index of this nomogram was 0.66 (95% CI: 0.64–0.68). The AUC of the nomogram was 0.67 (**Figure S3B**).

### Survival analysis

**Figure 3:**
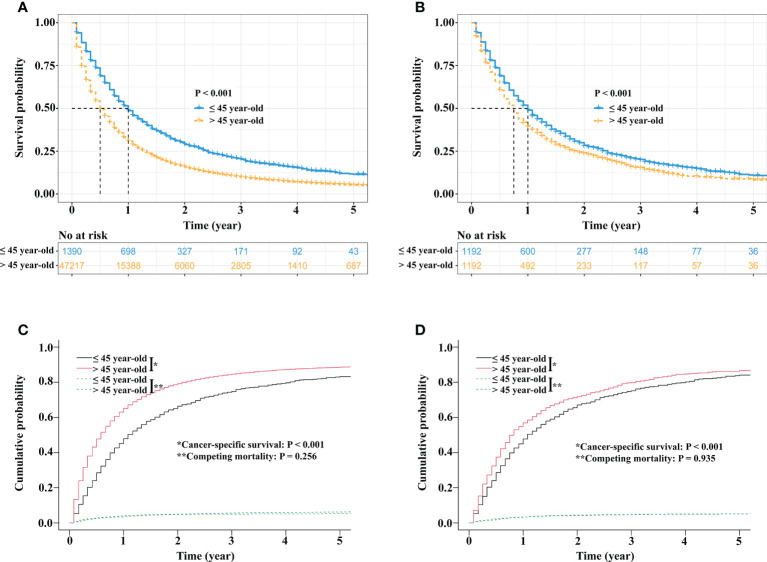
Survival comparisons between stage IV NSCLC patients aged ≤45 years and stage IV NSCLC patients aged >45 years. Kaplan−Meier survival curve comparison before PSM **(A)**. Kaplan−Meier survival curve comparison after PSM **(B)**. Competing risk analyses before PSM **(C)** and competing risk analyses after PSM **(D)**. NSCLC, non-small cell lung cancer; PSM, propensity score matching.

Before PSM, survival analysis showed that stage IV NSCLC patients ≤45 years had better OS than patients >45 years (3-year OS rate, 20.3% vs. 9.8%, *p* < 0.001; **Figure 3A**). In the matched cohort, the OS rate of stage IV NSCLC patients ≤45 years was still superior to that of stage IV NSCLC patients >45 years (3-year OS rate, 20.3% vs. 14.6%, *p* < 0.001; **Figure 3B**).

Regarding CSS, before PSM, the 3-year cancer-specific cumulative mortality rate of stage IV NSCLC patients ≤45 years was lower than that of stage IV NSCLC patients >45 years (74.8% vs. 84.6%, *p* < 0.001; **Figure 3C**). After PSM, stage IV NSCLC patients ≤45 years still had better CSS than stage IV NSCLC patients >45 years (3-year cancer-specific cumulative mortality rate, 74.9% vs. 80.9%, *p* < 0.001; **Figure 3D**).

## Discussion

This large population-based study was the first to investigate the incidence, presentations, and survival outcomes of stage IV NSCLC among young patients. The findings of this study can be summarized as follows. The incidence of stage IV NSCLC patients ≤45 years has declined over the years. Stage IV NSCLC patients ≤45 years were more likely to be diagnosed at an advanced stage, but they were also more likely to receive aggressive treatments. Multivariable Cox analyses revealed that receiving surgery and chemotherapy were important prognostic factors. Survival analyses showed that the survival of younger stage IV NSCLC patients was superior to that of the older patients. Our study focused on a special subset of stage IV NSCLC patients, which may provide comprehensive knowledge of this population.

There are conflicting data about the incidence of NSCLC among young individuals. Several studies have demonstrated that the incidence rate has been increasing gradually (5–7). Another study revealed that the incidence decreased from 1978 to 2010 (15). However, there are no studies that specifically investigated the incidence of stage IV NSCLC among young individuals. Our results showed a decreasing incidence of stage IV NSCLC among young individuals ≤ 45 years between 2004 and 2015. In our view, the reduced incidence might be attributed to economic development and improved living standards. Over the past few decades, poverty and geographical restraint have prevented some NSCLC patients from receiving standard cancer care services (9). Therefore, many NSCLC patients are diagnosed at advanced stages on initial examinations. With the advancement of diagnostic and treatment modalities, many patients are well managed with surgery and other novel efficient therapies in the early stage. In addition, less indoor air pollution and less exposure to coal dust might be partially responsible for the decline in incidence.

Consistent with previous studies (4, 5, 9–12, 14–16, 23), we also found that more young NSCLC patients were diagnosed with the ADC histology subtype. A possible explanation might be related to smoking: it is known that smoking is considered an important carcinogenesis factor for SCC (24, 25), and young patients are unlikely to be smokers (9, 13, 23). Therefore, the ADC histology subtype dominated in this population. No sex differences were observed in our study, which was similar to several other studies (4, 9, 23). Our study showed that when compared with the older cohort, younger patients were more likely to be diagnosed at advanced stages and receive aggressive treatments. This result was also confirmed by Arnold et al. (26), who demonstrated that young patients were administered more aggressive therapies than older patients at each TNM stage. Better performance status and a stronger desire to live may explain this difference.

Controversy exists with regard to the survival differences between younger and older stage IV NSCLC patients. A study by Vashistha et al. demonstrated that the survival of young NSCLC patients is similar to that of older NSCLC patients in India (9). This was also confirmed in a study by Mauri et al. (14). However, Bratova et al. (10) and Bryant et al. (13) suggested that young NSCLC patients have worse survival rates than older NSCLC patients. Our study showed that the survival of stage IV NSCLC patients ≤45 years was superior to that of stage IV NSCLC patients >45 years. Similar findings were also observed in the studies by Arnold et al. (26) and Subramanian et al. (12). One potential reason for this difference was that young patients were more likely to undergo surgery and chemotherapy than older patients. Previous clinical series suggested that surgical resection could prolong stage IV patient survival (27–32). Our nomograms also confirmed that surgery was the strongest predictor of favorable outcomes, followed by chemotherapy and the T category. Therefore, it is reasonable to observe that young patients had better survival outcomes than older patients. Additionally, a better performance status might bring survival benefits to young patients. Herein, we proposed that a multidisciplinary collaborative treatment modality, including surgery, might be the preferred option for these patients with advanced diseases.

Our study had several limitations. First, we could not evaluate the influence of performance status, smoking history, comorbidities, timing, dosage and regimens of treatments, and treatment-related side effects because they were not recorded in the SEER database. It is likely that with increasing age, more comorbidities and worse performance status might hinder older patients from receiving more aggressive treatments. Second, in the era of targeted therapy and immunotherapy, older stage IV NSCLC patients could gain more survival benefit from novel efficient therapies. However, genetic features such as epidermal growth factor receptor mutation status, anaplastic lymphoma kinase mutation status, programmed death ligand 1 expression level, and tumor mutation burden were not recorded in the database. Further efforts on broader clinicopathological features such as radiomics features (33) and tumor molecular profiles (34) recruitment are also warranted. Third, external validation was lacking in this study. Therefore, our results needed to be further validated in other clinical series and should be cautiously interpreted. Finally, inevitable bias was inherent to the retrospective design of the study, although PSM was used in the study.

Taken together, the incidence of stage IV NSCLC patients aged ≤45 years declined over the years. Although patients in this cohort were more likely to be diagnosed at advanced stages, they were also more likely to receive aggressive treatments. The prognosis of the young patients was better than that of the older patients. Although the younger cohort had better survival outcomes, they deserved more attention due to their young age and the significant socioeconomic implications of disease among this group.

## Data availability statement

The original contributions presented in the study are included in the article/**Supplementary Material**. Further inquiries can be directed to the corresponding authors.

## Ethics statement

The studies involving human participants were reviewed and approved by Institutional Review Board of Peking University People’s Hospital. Written informed consent for participation was not required for this study in accordance with the national legislation and the institutional requirements.

## Author contributions

FY and XW contributed to conception and design of the study. J-SC and M-TQ organized the database. J-SC performed the statistical analysis. J-SC wrote the first draft of the manuscript. J-SC wrote sections of the manuscript. All authors contributed to manuscript revision, read, and approved the submitted version.

## Funding

This work was supported by the National Natural Science funds (grant number 82173386).

## Conflict of interest

The authors declare that the research was conducted in the absence of any commercial or financial relationships that could be construed as a potential conflict of interest.

## Publisher’s note

All claims expressed in this article are solely those of the authors and do not necessarily represent those of their affiliated organizations, or those of the publisher, the editors and the reviewers. Any product that may be evaluated in this article, or claim that may be made by its manufacturer, is not guaranteed or endorsed by the publisher.

## Supplementary material

Supplementary Figure 1 Prognostic predictor selection using the LASSO regression analysis. LASSO coefficient profiles of 18 variables against the log (Lambda) sequence for OS **(A)** and CSS **(C)**. Tuning parameter (Lambda) selection in the LASSO model used 10-fold cross-validation *via* minimum criteria for OS **(B)** and CSS **(D)**. LASSO, least absolute shrinkage and selection operator; OS, overall survival; CSS, cancer specific survival.

Supplementary Figure 2 Prognostic nomograms for OS **(A)** and CSS **(B)**. OS, overall survival; CSS, cancer specific survival; ADC, adenocarcinoma; SCC, squamous cell carcinoma.

Supplementary Figure 3 The receiver operating curves of the nomograms. **(A)** OS and **(B)** CSS. AUC, area under curve; OS, overall survival; CSS, cancer specific survival.
