# Supplementary figures and images for: Stage IV non-small cell lung cancer among young individuals: Incidence, presentations, and survival outcomes of conventional therapies
Source: Front Oncol. 2022 Nov 11;12:894780. doi: 10.3389/fonc.2022.894780 (PMC9691661; doi:10.3389/fonc.2022.894780)

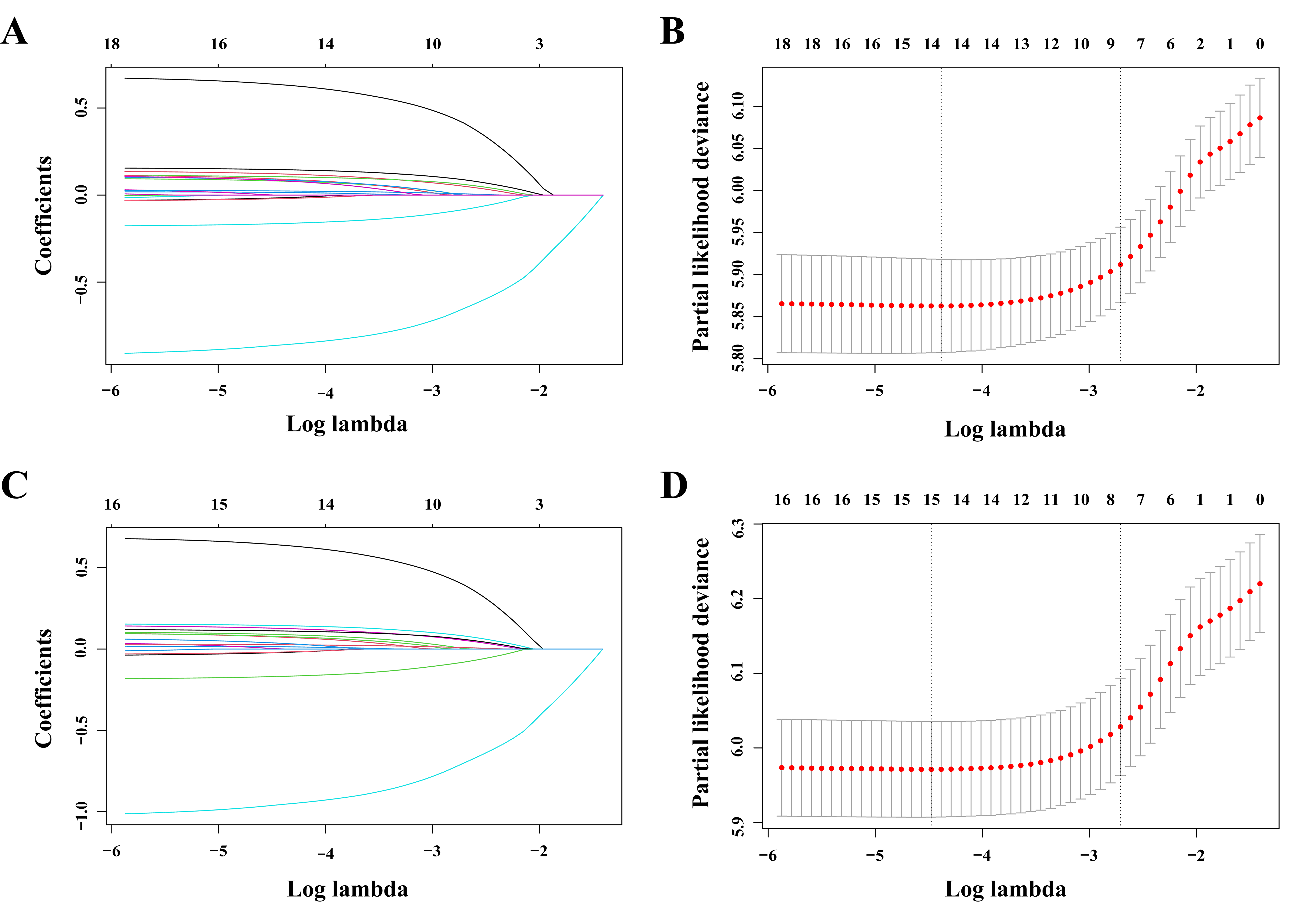

Supplement: Supplementary Figure 1 — Prognostic predictor selection using the LASSO regression analysis. LASSO coefficient profiles of 18 variables against the log (Lambda) sequence for OS (A) and CSS (C). Tuning parameter (Lambda) selection in the LASSO model used 10-fold cross-validation via minimum criteria for OS (B) and CSS (D). LASSO, least absolute shrinkage and selection operator; OS, overall survival; CSS, cancer specific survival. [file Image_1.tif]

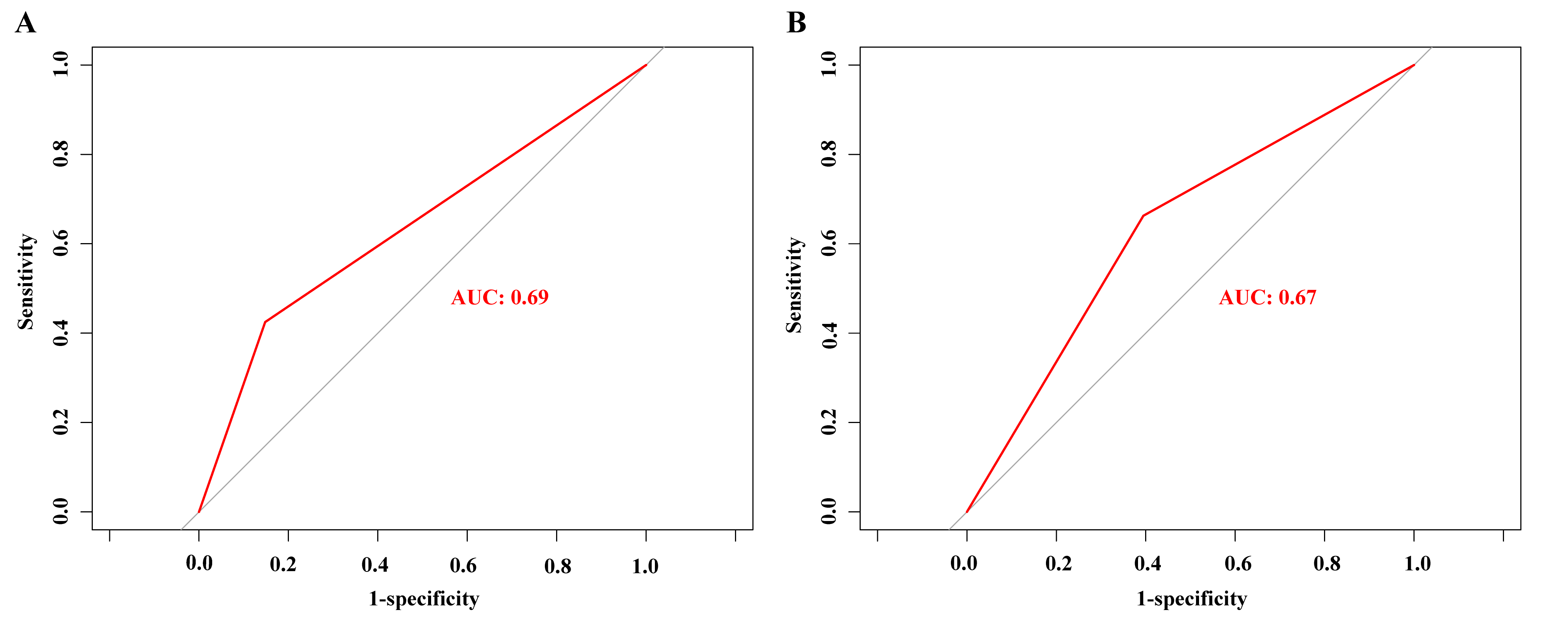

Supplement: Supplementary Figure 3 — The receiver operating curves of the nomograms. (A) OS and (B) CSS. AUC, area under curve; OS, overall survival; CSS, cancer specific survival. [file Image_3.tif]
